# Combined Bouveret and Type IV Mirizzi Syndromes Treated by One‐Stage Biliary and Gastrointestinal Reconstruction: A Case Report

**DOI:** 10.1155/cris/6543672

**Published:** 2026-10-25

**Authors:** Madeline Ryu, Ching-Kuo Chang, Sidney T. Le

**Affiliations:** ^1^ Department of Surgery UC San Francisco East Bay Oakland California USA; ^2^ Department of Surgery Kaiser Permanente Oakland Oakland California USA

**Keywords:** Bouveret, case report, Mirizzi’s, one-stage approach

## Abstract

Bouveret syndrome is a rare condition that occurs due to migration of a gallstone into the duodenum through fistula formation between the gallbladder and duodenum, leading to gastric outlet obstruction. Mirizzi’s syndrome is a rare condition where cholelithiasis leads to external compression or even erosion into the common hepatic duct (CHD). Here, we present a unique case that showcases severe presentations of both phenomena in a male presenting with sudden‐onset pain and vomiting for 3 h. The decision for a one‐stage approach of fistula takedown, cholecystectomy, duodenectomy, Roux‐en‐Y, and hepaticojejunostomy was made given the patient’s young age and good health status after failure of stone retrieval through esophagogastroduodenoscopy (EGD). Beyond anatomic necessity, simultaneous stone clearance and cholecystectomy avoid the recurrent lithiasis, interval cholecystitis, and ongoing erosion into adjacent structures associated with a staged approach. This case underscores that operative planning should be guided by anatomic complexity rather than age or ASA class alone, provided patient selection is appropriate.

## 1. Introduction

The presentation of cholelithiasis is rarely seen as gallstone erosion into adjacent organs. The incidence of cholecystoduodenal fistulas on presentation is cited as anywhere from 0.3% to 0.5% of all gallstone presentations [1]. Gallstone ileus presents with the typical symptoms of small bowel obstruction (i.e., nausea, vomiting, abdominal distension, and pain) and is sometimes associated with or preceded by cholecystitis/cholelithiasis symptoms. This is usually due to a large obstructing stone at the ileocecal junction [1]. Bouveret syndrome presents in a subset of cholecystoduodenal fistulas and is characterized by stone impaction in the pylorus or proximal duodenum, leading to gastric outlet obstruction. Bouveret syndrome occurs in only 2%–3% of all gallstone ileus presentations, which itself is only 1%–4% of all presentations of all small bowel obstructions [2, 3]. Mirizzi’s syndrome occurs due to extrinsic compression of a gallstone onto the common bile duct (CBD) and in severe cases can result in erosion into the CBD. Csendes classification defines Mirizzi’s into four types. Type 1 is solely external compression of the CBD by a stone in the infundibulum of the gallbladder. Type 2 Mirizzi is when an actual fistula is created but only involves ⅓ of the CBD. Type 3 involves ⅔ of the CBD, and Type 4 is when the CBD is no longer recognizable or distinguishable from the gallbladder [4]. Our patient presents with both Bouveret and Mirizzi’s syndromes.

However, in addition to Bouveret’s syndrome, this patient had complete degeneration/degradation of the entire anterior wall of the common hepatic duct (CHD). There are many hypotheses for bile duct perforations, including “calculous perforation at the site of impaction; calculous erosion without impaction; increased canalicular pressure due to obstruction by tumor, stone, or spasm of the sphincter of Oddi; intramural infection; mural vessel infarction leading to mural necrosis; or rupture of a biliary tract anomaly such as cyst or diverticulum” [5]. Mirizzi syndrome commonly presents with jaundice due to extrinsic compression of the CBD by impacted stones within the gallbladder [6].

We are presenting a unique case that was found to have Bouveret’s and Mirizzi’s syndromes due to the heavy stone burden. However, this is not a typical case of Mirizzi/Bouveret’s due to the complete erosion of the CHD causing fistulous connections of the duodenum‐CHD‐gallbladder‐CBD. Although variations in biliary anatomy are well recognized, this specific configuration of complete erosion of the CHD due to gallstone burden is an anatomic variant that has been rarely described in the literature. Moreover, we present a successful management option with a one‐step approach for cases like these. The patient was fully informed of the material to be published and provided written consent accordingly. This case report was reviewed by the Institutional Review Board of Kaiser Permanente Northern California, which determined that this study is exempt from formal ethical review.

## 2. Case Presentation

A 55‐year‐old man presented with 3 h of acute‐onset pain after eating. The patient reported inability to tolerate oral intake due to ongoing nausea and vomiting. The patient stated that he had 3 years of pain after eating but thought this was due to his reflux symptoms, which he has had for years. Work‐up started with a full set of labs, which included liver function tests, which were all normal: Alk phosphatase 76 U/L (reference range 44–147 U/L), AST 26 U/L (10–40 U/L), ALT 20 U/L (7–56 U/L), and Tbili 0.6 mg/dL (0.2–1.2 mg/dL). Further lab evaluations were also all normal: lipase 28 U/L (0–160 U/L), WBC 8.5 × 10^9^/L (reference range 4.0–11.0 × 10^9^/L), Hgb 14.8 g/dL (13.5–17.5 g/dL), Hct 44% (41%–53%), and platelet 235 × 10^9^/L (150–450 × 10^9^/L). Please refer to Table 1 for lab results.

**Table 1:** Laboratory values.

Lab value	Result	Reference range
Alkaline phosphatase	76 U/L	44–147 U/L
AST	26 U/L	10–40 U/L
ALT	20 U/L	7–56 U/L
Total bilirubin	0.6 mg/dL	0.2–1.2 mg/dL
Lipase	28 U/L	0–160 U/L
WBC	8.5 × 10^9^/L	4.0–11.0 × 10^9^/L
Hemoglobin	14.8 g/dL	13.5–17.5 g/dL
Hematocrit	44%	41%–53%
Platelets	235 × 10^9^/L	150–450 × 10^9^/L

Dissection was initially considered by the emergency physicians and was first ruled out with chest X‐ray (XR) and bedside ultrasound (US). Also on the differential with these symptoms would be symptomatic cholelithiasis, cholecystitis, and cholangitis (though white blood count was normal). Right upper quadrant (RUQ) US was done showing cholelithiasis, normal CBD, and no cholecystitis. The stone burden on RUQ US was not remarkable as this was only able to see the stones within the gallbladder itself and not in the duodenum or stomach. It commented on intrahepatic biliary dilation but could not see the extrahepatic biliary anatomy. The patient’s pain had initially improved, so he was initially arranged for outpatient cholecystectomy, but pain worsened again, so he was taken to CT scan.

CT scan (Figures 1 and 2) revealed a markedly dilated stomach, a fistulous connection between the proximal duodenum or distal stomach and the gallbladder, and possible erosion of the gallbladder into the CHD.

**Figure 1:**
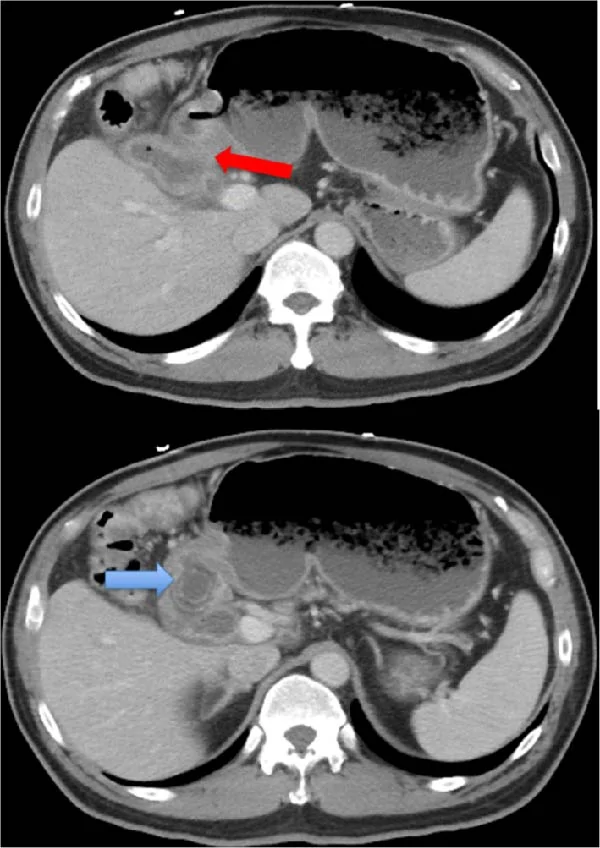
Cholecystoduodenal fistula on CT axial. Red arrow indicates fistulous connection between gallbladder and pylorus/duodenum with thickened walls. Blue arrow indicates gallstone eroded into the pyloric lumen with thickened gastric/duodenal wall.

**Figure 2:**
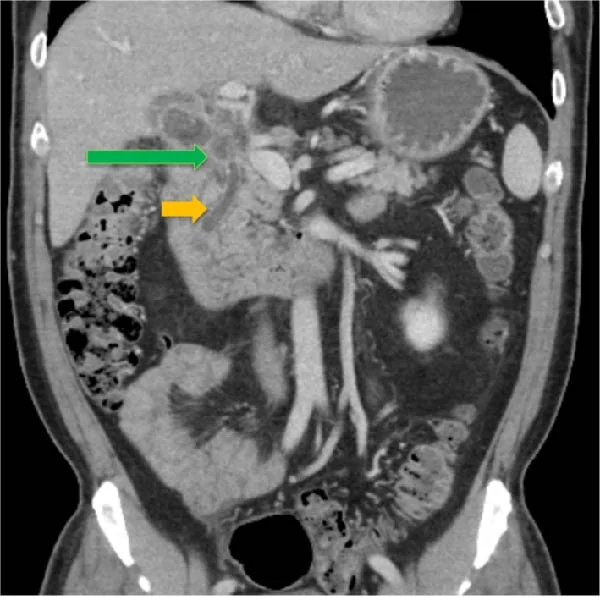
Cholecystoduodenal fistula on CT coronal. Orange arrow indicates dilated common bile duct (CBD). Green arrow indicates gallstone erosion and common channel of the common hepatic duct, gallbladder, and CBD.

Gastroenterology was consulted, and on hospital day (HOD) 1, he was taken for an attempted endoscopic retrieval of the gallstone, which was unsuccessful due to a very large stone lodged just distal to the pylorus (Figure 3). Multiple attempts were made with a rat tooth, talon, snare, and Roth Net, but the stone was extremely firm and the scope was unable to traverse around it.

**Figure 3:**
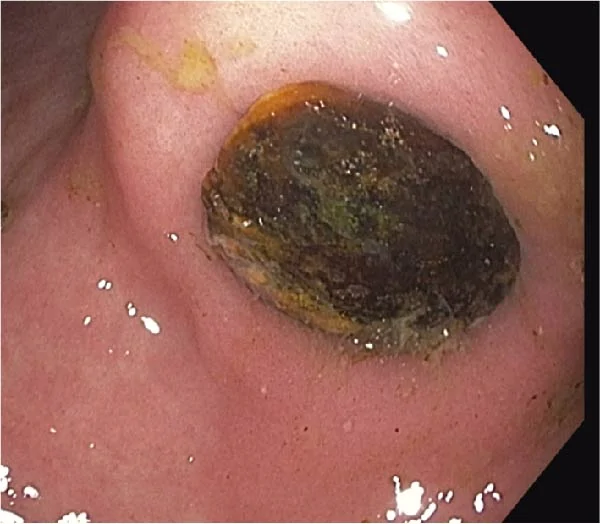
Impacted stone on EGD.

Given that the patient had few comorbidities and good baseline functional status prior to presentation, and radiographic evidence of heavy stone burden that was relatively large in size (up to 3 cm), which would continue to cause repeat episodes of obstruction, the decision was made to proceed with surgical exploration, which included a one‐stage approach of CBD exploration, stone extraction, fistula takedown, and cholecystectomy (or as much removed as safely as possible).

A right subcostal incision was utilized to enter the abdomen. The duodenum was Kocherized in order to mobilize the inflamed tissue and more clearly see the structures under question. Immediately, severe adhesions were encountered surrounding the gallbladder and the fistula. The lateral and anterior gallbladder walls were excised, and several large gallstones within the gallbladder were removed. Upon further inspection, it was seen that the gallbladder had eroded and fistulized to the CHD. This was confirmed by passing a Fogarty catheter distally, which also allowed us to confirm the location of the ampulla, which was distal to the cholecystoduodenal fistula. Furthermore, the CBD was no longer identifiable, which is defined as Type IV Mirizzi, as the stones had completely eroded through the CBD and the CHD. Due to the completely open nature of the CHD and CBD at this point, we could not simply remove the stone and close as the two‐step approach would entail. We therefore opted to perform an antrectomy/proximal duodenectomy that included the removal of the stone that was causing gastric outlet obstruction. We then created a roux‐en‐Y hepaticojejunostomy to reconstruct the biliary system and a gastrojejunostomy to reestablish continuity. A drain was left in place at the hepaticojejunostomy anastomosis. The patient had an uneventful postoperative course, and the drain, which was nonbilious, was removed on post‐op day 9. The patient has made a full recovery when an in‐person clinic visit was done 6 months after the operation and is back to his normal living. The patient reports a return to normal life and denies any gastrointestinal disturbances, including dyspepsia or abdominal pain, to date. Given his favorable recovery, no additional workup was pursued. His sole reported symptom is numbness localized to the laparotomy incision site; he otherwise reports a satisfactory postoperative recovery.

## 3. Discussion

Bouveret syndrome is rare and diagnostically challenging. Rigler’s Triad—pneumobilia, gastric distension, and ectopic radio‐opaque calculi—is pathognomonic but present in only 14%–53% of plain radiographs, 11% of USs, 80% of CT scans, and 100% of MRI studies [7, 8]. Mirizzi is also exceedingly difficult to diagnose, as there is a low index of suspicion for this condition among surgeons due to its rarity [9]. Our patient lacked the typical demographic profile (elderly female, large antecedent gallstones, or prior altered surgical anatomy), which further delayed recognition and underscores that clinical suspicion must not be confined to the classic presentation.

Within the literature, the initial management of Bouveret syndrome is focused on endoscopic or surgical stone extraction. However, it is relatively common for esophagogastroduodenoscopy (EGD) to be insufficient in management and extraction of these stones due to the sheer size of the stone causing such an obstruction. Generally, a stone greater than 2.5 cm in diameter will cause an obstruction in the intestinal tract with a 91% failure rate to extract stones endoscopically [2]. It has been reported that around 73% of patients undergo surgery for Bouveret syndrome [10]. EGD reveals gastroduodenal obstruction in 100% of cases but was only able to identify the obstructing stone in 69% of cases and the fistula itself in 13% of cases [11]. The complications of an EGD are not benign and can result in stone impaction in the esophagus, gallstone ileus, hemorrhage, or perforation [3]. When endoscopic stone extraction is unsuccessful, it has been advocated to proceed with surgical stone extraction, which is typically performed by milking the stone proximally and performing a gastrotomy. Our patient also proved to have a stone that was unextractable with EGD and was forced to proceed with surgery to relieve this obstruction, which brings us to the discussion of the operative choice and decisions made for this case.

Preoperative delineation of the relationship between the gallbladder, the CBD, and adjacent viscera proved critical in this case. Failure to define this anatomy carries significant operative risk as the findings directly dictate the reconstructive strategy. While US and CT imaging were performed, magnetic resonance cholangiopancreatography (MRCP) was considered to further characterize the biliary anatomy. In this clinical context, however, MRCP would not have changed anything as we had a high suspicion of CBD disruption on CT and EGD findings. The plan was to perform a CBD exploration, which is what we did in the operating room. EGD with possible ERCP was favored as it offered both direct assessment of the anatomy and the potential for immediate therapeutic intervention. Additionally, MRCP availability can vary across institutions, which may influence the feasibility of obtaining this imaging modality in certain settings. Moreover, we found intraoperative use of a Fogarty balloon catheter invaluable: palpation of the inflated balloon confirmed fistulization of the gallbladder into the CHD, a finding that altered the planned procedure. The placement of a stent via ERCP can help simplify the procedure with a better‐defined anatomy due to a stricture being found in all cases of those with Mirizzi [11, 12]. In select cases where inflammatory changes completely change the biliary anatomy, this one‐stage technique provides a demonstrated safe and successful option to facilitate safe operative management. Our patient was found to have Type IV Mirizzi syndrome—in which the CBD is incorporated into or replaced by the gallbladder wall—with the additional complexity of erosion into both the CHD and the duodenum. This combination is exceedingly rare and has no established management guidelines. The CHD involvement precluded simple fistula takedown and mandated biliary reconstruction; the duodenal erosion necessitated a bypass. Accordingly, we performed a one‐stage cholecystectomy with Roux‐en‐Y hepaticojejunostomy and gastrojejunostomy. The patient recovered without biliary complications.

The debate between one‐stage (stone extraction with concurrent cholecystectomy and fistula repair) and two‐stage (stone extraction followed by interval cholecystectomy) approaches remains unresolved in the literature [13, 14]. Proponents of the two‐stage approach state that because most patients who present with gallstone ileus are elderly with poor health or many comorbidities, the risks of the one‐stage are too high, and performing a minimally invasive surgery with shorter recovery times is more important and beneficial to this patient population. They also cite that fistulas may self‐resolve once the offending stone has been removed and the cystic duct is freely flowing [15, 16]. Following endoscopic or surgical stone extraction, cholecystectomy and cholecystoduodenal fistula takedown can be performed at a later date.

Supporters of the one‐stage approach state that one‐stage prevents significant postoperative morbidity by preventing subsequent biliary complications such as cholecystitis, cholangitis, and prevention of possible gallbladder cancer [17]. Lastly, some are pushing for stone removal or enterolithotomy alone without any further procedures for a select population. Proponents of this approach have quoted studies showing increased perioperative morbidity and mortality rates when comparing the one‐stage approach with enterolithotomy alone [14, 18].

In this case, the anatomy compelled a one‐stage approach—the CHD erosion required immediate reconstruction, and staged repair would have left the patient with an unreconstructed biliary‐enteric fistula. With Type IV Mirizzi, it is well accepted that the best surgical approach would be a cholecystectomy and Roux‐en‐Y hepaticojejunostomy [9, 19, 20]. Beyond the anatomical imperative, we believe that complete stone clearance and cholecystectomy performed simultaneously eliminates the risks of recurrent lithiasis, interval cholecystitis, and progressive erosion into adjacent structures that attend the two‐stage strategy. Careful patient selection remains essential, but this case demonstrates that anatomic complexity, rather than age or ASA class alone, should drive the operative decision.

As minimally invasive techniques continue to evolve, individualized assessment—incorporating patient physiology, surgeon experience, and intraoperative anatomic findings—will be increasingly important. When biliary anatomy is distorted, systematic intraoperative assessment of the fistula’s relationship to the ampulla of Vater, CHD, gallbladder, CBD, and duodenum is essential before any reconstructive commitment is made.

## Funding

No funding was received for this study.

## Conflicts of Interest

The authors declare no conflicts of interest.

## Data Availability

The data that support the findings of this study are available from the corresponding author upon reasonable request.
